# Molecular basis for the recognition of cyclic-di-AMP by PstA, a P_II_-like signal transduction protein

**DOI:** 10.1002/mbo3.243

**Published:** 2015-02-18

**Authors:** Philip H Choi, Kamakshi Sureka, Joshua J Woodward, Liang Tong

**Affiliations:** 1 Department of Biological Sciences, Columbia UniversityNew York City, New York, 10027; 2 Department of Microbiology, University of WashingtonSeattle, Washington, 98195

**Keywords:** Crystal structure, cyclic-di-AMP, Firmicutes, *Listeria monocytogenes*, P_II_-like protein, signal transduction

## Abstract

Cyclic-di-AMP (c-di-AMP) is a broadly conserved bacterial second messenger that is of importance in bacterial physiology. The molecular receptors mediating the cellular responses to the c-di-AMP signal are just beginning to be discovered. PstA is a previously uncharacterized P_II_-like protein which has been identified as a c-di-AMP receptor. PstA is widely distributed and conserved among Gram-positive bacteria in the phylum Firmicutes. Here, we report the biochemical, structural, and functional characterization of PstA from *Listeria monocytogenes*. We have determined the crystal structures of PstA in the c-di-AMP-bound and apo forms at 1.6 and 2.9 Å resolution, respectively, which provide the molecular basis for its specific recognition of c-di-AMP. PstA forms a homotrimer structure that has overall similarity to the P_II_ protein family which binds ATP. However, PstA is markedly different from P_II_ proteins in the loop regions, and these structural differences mediate the specific recognition of their respective nucleotide ligand. The residues composing the c-di-AMP binding pocket are conserved, suggesting that c-di-AMP recognition by PstA is of functional importance. Disruption of *pstA* in *L. monocytogenes* affected c-di-AMP-mediated alterations in bacterial growth and lysis. Overall, we have defined the PstA family as a conserved and specific c-di-AMP receptor in bacteria.

## Introduction

Bacteria utilize a wide array of nucleotide-based signaling molecules. Cyclic-di-AMP (c-di-AMP) is a broadly conserved and functionally diverse cyclic-dinucleotide second messenger that is found in many bacterial species. The levels of c-di-AMP in the cell are controlled through the opposing activities of diadenylate cyclases (DACs) which synthesize c-di-AMP from two molecules of ATP or ADP (Witte et al. 2008; Bai et al. 2012) and DHH/DHHA1 or HD domain containing phosphodiesterases which degrade c-di-AMP to pApA or AMP (Rao et al. 2010; Bai et al. 2013; Huynh et al. 2015). Cyclic-di-AMP is of fundamental importance for microbial physiology including bacterial growth and metabolism, cell morphology, potassium homeostasis, stress responses, antibiotic resistance, and virulence (Corrigan et al. 2011; Oppenheimer-Shaanan et al. 2011; Luo and Helmann 2012; Pozzi et al. 2012; Smith et al. 2012; Corrigan and Grundling 2013; Mehne et al. 2013; Campos et al. 2014; Sureka et al. 2014).

Several macromolecules such as the metabolic enzyme pyruvate carboxylase (Sureka et al. 2014), the *ydaO* riboswitch family (Nelson et al. 2013), the transcription factor DarR (Zhang et al. 2013), and proteins involved in potassium uptake (Corrigan et al. 2013; Bai et al. 2014) have been identified as c-di-AMP receptors in bacteria. In addition, the innate immune system in humans can detect secreted c-di-AMP during bacterial infection through the cytosolic surveillance protein STING (stimulator of interferon genes), leading to a host type I interferon response (Woodward et al. 2010; Burdette and Vance 2013). Recent studies on c-di-AMP signaling have begun to unravel some of the molecular mechanisms through which c-di-AMP exerts its physiological effects in bacteria. Crystal structures of c-di-AMP bound to the metabolic enzyme pyruvate carboxylase (Sureka et al. 2014) and the *ydaO* riboswitch (Gao and Serganov 2014; Jones and Ferre-D'Amare 2014; Ren and Patel 2014) have provided insights into the mechanism by which c-di-AMP is recognized by its receptors.

**Figure 1:**
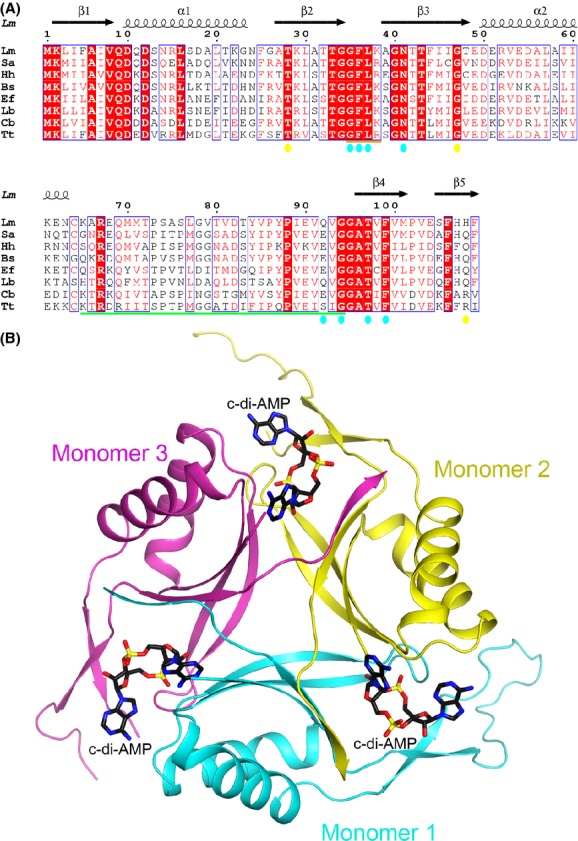
Crystal structure of *Listeria monocytogenes* PstA in complex with cyclic-di-AMP (c-di-AMP). (A) Sequence alignment of PstA from *L. monocytogenes* (Lm), *Staphylococcus aureus* (Sa), *Halobacillus halophilus* (Hh), *Bacillus subtilis* (Bs), *Enterococcus faecalis* (Ef), *Lactobacillus brevis* (Lb), *Clostridium botulinum* (Cb), and *Thermoanaerobacterium thermosaccharolyticum* (Tt). The secondary structure elements are indicated at the top of the alignment. Conserved residues are indicated in red. Residues that interact with c-di-AMP are colored cyan (monomer 1) or yellow (monomer 2). The T-loop is indicated by the orange bar. The B-loop is indicated by the green bar. Produced with Espript (Gouet et al. 1999). (B) Schematic drawing of the LmPstA homotrimer in complex with three c-di-AMP molecules. The three monomers are colored separately and the c-di-AMP molecules are labeled. The structure figures were produced in PyMOL (http://www.pymol.org).

PstA (P_II_-like signal transduction protein A) is a previously uncharacterized protein that has been reported to bind c-di-AMP in *Staphylococcus aureus* (Corrigan et al. 2013) and *Listeria monocytogenes* (Sureka et al. 2014), and its sequence is highly conserved among the collection of bacterial species (Fig.1A). PstA contains a DUF970 domain which is predicted to be structurally homologous to the nitrogen regulatory P_II_ proteins (Corrigan et al. 2013). P_II_ proteins are able to sense ATP, ADP, and 2-oxoglutarate levels in the cell (Corrigan et al. 2013; Huergo et al. 2013), and these various ligands modify the P_II_ structure promoting its binding and regulation of protein targets such as the ammonium channel AmtB (Conroy et al. 2007; Gruswitz et al. 2007). There have been many reported crystal structures of P_II_ proteins bound to its ligands, and the binding pocket for ATP has been particularly well characterized (Xu et al. 1998; Sakai et al. 2005; Shetty et al. 2010). In contrast, PstA was found to bind specifically to c-di-AMP but not ATP (Sureka et al. 2014), although the structural basis for this selectivity was not known. Here, we report the crystal structures of PstA from *L. monocytogenes* (LmPstA) bound to c-di-AMP and in the apo form. A comparison of these structures to the ATP-bound P_II_ proteins reveals significant differences in the architecture of the binding pocket for each ligand, providing a structural basis for the specific binding of c-di-AMP to PstA. We also find that the c-di-AMP binding pocket in PstA is highly conserved (Fig.1A), suggesting that this interaction is of importance in bacterial physiology.

## Experimental Procedures

### Protein expression and purification

*L. monocytogenes* pstA (lmo2692) was subcloned into a pET20b vector with a C-terminal His-tag. This construct was transformed into BL21(DE3) Rosetta cells. The cells were cultured in LB (Luria–Bertani) medium with 35 mg/L kanamycin and 35 mg/L chloramphenicol and were induced for 14 h with 1 mmol/L Isopropyl *β*-D-1-thiogalactopyranoside at 20°C. The protein was purified through nickel-agarose affinity chromatography followed by gel filtration chromatography (S-300, GE Healthcare, Piscataway, New Jersey, USA). The purified protein was concentrated to 20 mg/mL in a buffer containing 20 mmol/L Tris (pH 8.0), 150 mmol/L NaCl, 5% (v/v) glycerol, and 5 mmol/L dithiothreitol, flash-frozen in liquid nitrogen and stored at −80°C. The C-terminal hexa-histidine tag was not removed for crystallization.

### Crystallization

LmPstA crystals were grown by the sitting-drop vapor diffusion method at 20°C. For the c-di-AMP complex, the protein at 20 mg/mL was incubated with 2.5 mmol/L c-di-AMP for 30 min at 4°C before setup. The reservoir solution contained 12% (w/v) PEG3350 and 0.1 mol/L sodium malonate (pH 4.0). The crystals appeared within 1–2 weeks. The crystals were cryoprotected in the reservoir solution supplemented with 15% (v/v) glycerol and were flash-frozen in liquid nitrogen for data collection at 100 K. For the apo LmPstA structure, the protein at 10 mg/mL was mixed with a reservoir solution contained 20% (w/v) PEG3350, 0.1 mol/L bis-Tris (pH 5.5), and 0.1 mol/L ammonium acetate. The crystals appeared within 1 week and grew to full size within a few more days. They were cryoprotected in the reservoir solution supplemented with 10% (v/v) glycerol and flash-frozen in liquid nitrogen.

### Data collection and structure determination

All X-ray diffraction data were collected at the X29 beamline at the National Synchrotron Light Source (NSLS) at Brookhaven National Laboratory. The diffraction images were processed using HKL3000 (Minor et al. 2006). The structures were solved using the molecular replacement method with the program Phaser (McCoy et al. 2008), using a PstA homolog from *Pediococcus pentosaceus* (PDB code 3M05) as the search model. Manual rebuilding was carried out in Coot (Emsley and Cowtan 2004) and refinement was done with the program Refmac (Murshudov et al. 1997). Coordinates and structure factors have been deposited in the Protein Data Bank with accession codes 4RWW and 4RWX.

### Site-directed mutagenesis

Mutants were made using the QuikChange kit (Agilent Technologies, Santa Clara, California, United States) and confirmed by sequencing. The primers used for generation of amino acid variants are listed in Table1. PstA containing point mutants were expressed and purified using the same protocol as the wild-type (WT) protein.

**Table 1:** Primers used in this study

Primer name	Sequence	Description
Δ*pstA* A *Sac*I fwd	GAGGAGGAGCTCGCGACCGAATTCGCATTAC	Primer for amplifying 1000 bp upstream of *pstA*. The *Sac*I cut site is underlined
Δ*pstA* B rev	TCCCACTATCTGTTCTAAAAATGATGGAGTTTCAAAGAAATCAACCCTTCC	Primer for amplifying 1000 bp upstream of *pstA*
Δ*pstA* C fwd	AGGAGGAAATGAAATGACGTTACGAATTTAATCGAGGAGGAACAAACTTTA	Primer for amplifying 1000 bp downstream of *pstA*
Δ*pstA* D *Pst*I rev	GAGGAGCTGCAGGGACTCACTTTGGAGAATCGC	Primer for amplifying 1000 bp downstream of *pstA*. The *Pst*I cut site is underlined
*pstA Kpn*I fwd	GTGGTGGTACCTTTTAGGAAGGGTTGATTTCTTTG	Primer for amplifying *pstA* into pJW282. The *Kpn*I cut site is underlined
*pstA Pst*I rev	GATTGCTGCAGCTAGTGATGATGATGATGATGAAAATGATGGAAACTCTCAA	Primer for amplifying *pstA* into pJW282. The *Pst*I cut site is underlined
*pstA* F36A S	GTTTCCTGCTTTTAAAGCTCCACCCGTTGTAGC	Sense primer for quick change mutagenesis of PstA
*pstA* F36A AS	GCTACAACGGGTGGAGCTTTAAAAGCAGGAAAC	Antisense primer for quick change mutagenesis of PstA
*pstA* N41A S	GATGATAAACGTGGTGGCTCCTGCTTTTAAAAA	Sense primer for quick change mutagenesis of PstA
*pstA* N41A AS	TTTTTAAAAGCAGGAGCCACCACGTTTATCATC	Antisense primer for quick change mutagenesis of PstA

### Construction of Δ*pstA* strain

Deletion of the chromosomal copy of *pstA* was accomplished using the pKSV7-*oriT* plasmid. Briefly, 1000 base pairs flanking the 5′ and 3′ ends of the *pstA* gene were amplified and subsequently combined by splicing by overlap extension (SOE) PCR using the primers described in Table1. A total of six amino acids from the original open reading frame were retained to limit disruption of downstream genes. The SOE amplified product was digested with *Sac*I and *Pst*I and ligated into similarly digested pKSV7-*oriT*. The plasmid was then conjugated into *L. monocytogenes* WT or cΔ*dacA* strains through the donor strain *E. coli* SM10. Subsequent selection for integration, plasmid excision, and plasmid curing were as described previously (Camilli et al. 1993).

### Complementation of Δ*pstA* strain

For complementation, *pstA* was expressed under the constitutive hyper-*Pspac* promoter. The *pstA* gene was amplified using the primers GTG GTG GTA CCT TTT AGG AAG GGT TGA TTT CTT TG and GAT TGC TGC AGC TAG TGA TGA TGA TGA TGA TGA AAA TGA TGG AAA CTC TCA A and the PCR product was digested with *Kpn*I and *Pst*I restriction enzymes and ligated into the plasmid pJW361 (Sureka et al. 2014) digested with the same enzymes. The resulting plasmid was then electroporated into different Δ*pstA* strains.

### Tissue culture and infection assays

Macrophage growth curves, DNA release, and plaque assays were performed in J2 immortalized marrow-derived macrophages (BMMs) (Sauer et al. 2010) or L2 mouse fibroblasts as indicated and described previously (Sun et al. 1990).

### Bacteriolysis and antibiotic susceptibility

Bacteriolysis as measured by *β*-galactosidase release and sensitivity to cefuroxime determined by the zone of inhibition in a disk diffusion assay were performed as described previously (Witte et al. 2013).

## Results

### Biochemical and genetic characterization of PstA

We previously identified LmPstA as a c-di-AMP binding protein utilizing a chemical proteomics approach (Sureka et al. 2014). It binds c-di-AMP specifically with a *K*_d_ of 1.4 *μ*mol/L, determined with DRaCALA (differential radial capillary action of ligand assay), which is consistent with the levels of c-di-AMP in bacterial cells (Corrigan et al. 2013). We analyzed the sequence database to assess the conservation of PstA in bacteria, and found that it is widely distributed in Firmicutes (low GC content Gram-positive bacteria), but is completely absent in Actinobacteria (high GC content Gram-positive bacteria) and Gram-negative bacterial species. The sequence of PstA consists of 109 residues and is generally well conserved between species (Fig.1A). LmPstA has 16% and 20% sequence identity with the *Escherichia coli* P_II_ proteins GlnK and GlnB, respectively.

We also analyzed the genomic context of the *pstA* gene in the bacterial species *L. monocytogenes*, *S. aureus*, *Bacillus subtilis*, *Enterococcus faecalis*, *Clostridium botulinum*, and *Lactobacillus brevis*. In all of these species, *pstA* is invariably found in an operon with the gene encoding thymidylate kinase, an enzyme involved in the purine salvage pathway. In several of these bacteria, a gene encoding an ornithine–arginine–lysine decarboxylase is also part of this operon.

### Overall structure of PstA and comparison with P_II_ proteins

We cocrystallized LmPstA with c-di-AMP and determined the structure of the complex at 1.6 Å resolution by the molecular replacement method (Fig.1B). A homolog of PstA from *P. pentosaceus* (PDB code 3M05), which shares 56% sequence identity with LmPstA, was used as the search model. We also determined the apo structure of LmPstA at 2.9 Å resolution. There is a trimer of LmPstA in the asymmetric unit for both crystals, and LmPstA migrated as a trimer on a gel filtration column. The final atomic models have good agreement with the X-ray diffraction data and the expected geometric parameters (Table2). All of the residues in both structures are in the favored region of the Ramachandran plot.

**Table 2:** Summary of crystallographic data

	c-di-AMP complex	apo
Space group	*P*1	*P*2_1_2_1_2_1_
Cell dimensions		
a, b, c (Å)	51.2, 51.8, 53.0	55.9, 61.6, 83.5
*α*, *β*, *γ* (°)	110.8, 105.6, 109.8	90, 90, 90
Resolution	40–1.6 (1.66–1.60)	40–2.9 (3.0–2.9)
*R*_merge_ (%)	9.0 (41.6)	6.1 (51.3)
I/*σ*I	15.8 (3.1)	22.4 (3.3)
Redundancy	3.9 (3.8)	4.7 (4.8)
Completeness	96.6 (94.6)	99.3 (100)
*R*_work_ (%)	15.7 (24.6)	22.4 (33.1)
*R*_free_ (%)	19.4 (30.2)	26.2 (45.2)
Average B-factors		
Protein	16.4	83.3
Ligand	11.3	–
Water	26.6	–
R.m.s. deviation bond lengths (Å)	0.015	0.011
R.m.s. deviation bond angles (°)	1.7	1.4
Ramachandran plot		
Favored	100%	100%
Outliers	0%	0%

**Figure 2:**
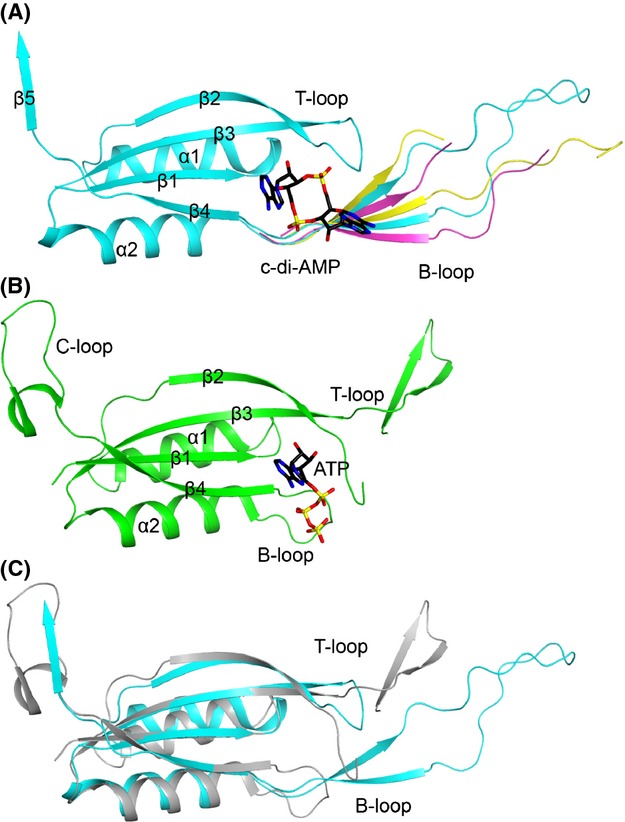
Structure of a PstA monomer and comparison with a P_II_ monomer. (A) Structure of a LmPstA monomer (cyan) in complex with cyclic-di-AMP (c-di-AMP) (black). The secondary structure elements and loops are labeled. The B-loops from the two other monomers are shown in yellow and pink. (B) Structure of a P_II_ monomer from *Mycobacterium tuberculosis* (green) in complex with ATP (black). (C) Overlay of the LmPstA monomer (cyan) with the P_II_ monomer (gray).

The structure of each LmPstA monomer is composed of a four-stranded antiparallel *β*-sheet packed against two *α*-helices on one face (Fig.2A). A fifth *β*-strand from a neighboring monomer of the trimer joins the *β*-sheet. We compared LmPstA to the crystal structure of a GlnK P_II_ protein bound to ATP from *Mycobacterium tuberculosis* (Mtb P_II_, PDB code 3LF0), which is structurally representative of the larger P_II_ family of proteins. The architecture of the LmPstA monomer is largely similar to the Mtb P_II_ monomer (Shetty et al. 2010) with a r.m.s. (root mean square) distance of 1.0 Å for their C*α* atoms when the monomers are overlaid without the loop regions (Fig.2B and C). However, several of the loops that connect the various *β*-strands and *α*-helices have notable differences. For consistency, the naming convention for the P_II_ protein loops will be used for the description of PstA. In Mtb P_II_, the T-loop (residues 36–55) is a 20-residue flexible extension located in the loop connecting strands *β*2 and *β*3. The B-loop (residues 82–87) is a smaller loop connecting *α*2 and *β*4 (Fig.2B). There is also a C-loop at the C-terminus of the protein. In PstA, however, the B-loop (residues 65–94) has a 30-residue extension while the T-loop (residues 35–38) is much shorter and LmPstA does not contain a C-loop (Figs.1A and 2A). The B-loop forms a twisted two-stranded *β*-sheet (residues 67–71 and 89–93) connected by a long looping turn (residues 72–88). The two-stranded *β*-sheet structure of the B-loop of LmPstA is similar to the T-loop of Mtb P_II_. However, the length of the turn connecting the two *β*-strands is much shorter in the Mtb P_II_ T-loop.

The three LmPstA monomers are almost identical in structure with an r.m.s. distance of 0.3 Å for their C*α* atoms, with the exception of the B-loop which shows variations among the three monomers, reflecting its flexibility (Fig.2A). Similarly, crystal structures of P_II_ proteins also show T-loops adopting various conformations (Sakai et al. 2005). However, the overall orientation of the LmPstA B-loop in relation to the core trimer structure is comparable between the three monomers (Fig.1B). The B-loop has several intermolecular interactions with the B-loops from neighboring trimers, so crystal contacts likely contribute to some degree to the observed conformations of this loop. A part of the B-loop in two of the monomers is disordered. In the apo LmPstA structure, all of the T-loops and B-loops have disordered regions.

In the LmPstA trimer, the *β*-sheet of the three monomers form the core, and the *α*-helices are on the periphery (Fig.1B). The overall organization of this trimer is similar to that of Mtb P_II_ and the r.m.s. distance is 0.9 Å for their C*α* atoms when the trimers are overlaid without the loop regions.

### The c-di-AMP binding mode in PstA

**Figure 3:**
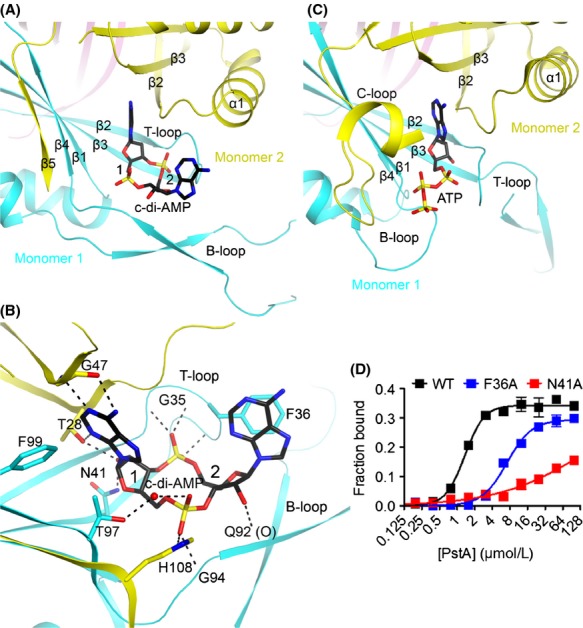
The PstA cyclic-di-AMP (c-di-AMP) binding pocket. (A) The overall organization of the LmPstA c-di-AMP binding pocket. The first and second nucleotide are labeled for c-di-AMP. (B) Detailed interactions between LmPstA and c-di-AMP. Hydrogen-bonding interactions are indicated with dashed lines (in black). Water molecules are shown as red spheres. (C) The overall organization of the Mtb P_II_ ATP binding pocket. (D) DRaCALA (differential radial capillary action of ligand assay) binding titration of c-di-AMP and LmPstA mutants using ^32^P-c-di-AMP.

Three c-di-AMP molecules are bound to symmetrically equivalent locations on the LmPstA homotrimer (Fig.1B), located in the inter-subunit cleft between *β*1 and *β*4, and the T/B-loops of one monomer and *α*1, *β*2–*β*3, and *β*5 of a neighboring monomer (Fig.3A). A fourth c-di-AMP molecule mediates contacts between LmPstA trimers in the crystal, which is likely a crystal-packing artifact and will not be discussed further.

The adenine base of the first nucleotide of c-di-AMP is recognized specifically by LmPstA, through hydrogen-bonds between its N1 and N6 atoms with the main chain amide and carbonyl of Gly47 (at the end of *β*3), respectively (Fig.3B). One face of this adenine is packed against the Gly26–Ala27 amide bond (near the beginning of *β*2), while the other face is in contact with the side chains of Thr97 and Phe99 (in *β*4) from the neighboring monomer. The 2′ OH of the ribose has hydrogen-bonding interactions with the side chains of Asn41 (*β*3 of neighboring monomer) and Thr28 (*β*2), with the latter also hydrogen-bonded to N3 of adenine. The phosphate group is hydrogen-bonded to the side chain His108 (*β*5) and the main chain amide of Gly94 (B-loop of neighboring molecule).

The binding site for this nucleotide of c-di-AMP in LmPstA is equivalent to that for ATP in Mtb P_II_ (Fig.3C). In contrast the recognition of the second nucleotide of the c-di-AMP molecule is mediated by unique structural features in PstA, due primarily to the significant structural differences in the T-loop which forms a part of the inter-subunit cleft where the ligand binds. The extended T-loop of Mtb P_II_ would clash with the adenine of the second nucleotide of c-di-AMP (Fig.3C). Instead, the T-loop in PstA forms a shorter, more structured loop with a conserved 34-GGFL-37 sequence in the turn. The main chain amides of Gly35, Phe36, and Leu37 have hydrogen-bonding interactions with the phosphate group of this nucleotide. The side chain of Phe36 is *π*-stacked against one face of the adenine, while its other face is exposed to the solvent.

In Mtb P_II_ structure, the B-loop and C-loop are important for anchoring the triphosphate moiety of ATP (Fig.3C). The B-loop has a highly conserved Walker A-like sequence motif (TGxxGDGKI) (Huergo et al. 2013) which interacts with the ATP triphosphate, and the C-loop also interacts with the ATP triphosphate through several arginine residues. In sharp contrast, PstA lacks the C-loop entirely, and the B-loop has a 30-residue flexible extension instead of the shorter, more structured B-loop found in P_II_ proteins. In addition, the B-loop of PstA does not contain a Walker A-like motif. As a result, the *β*- and *γ*-phosphates of ATP are unlikely to have strong interactions with PstA, which may explain why the protein does not bind this nucleotide.

To better characterize the c-di-AMP binding site in PstA, residues that were found to interact with c-di-AMP were substituted to alanine (Fig.3D). In particular, we mutated several conserved residues on the T-loop including Phe36 and Asn41. The F36A mutant exhibited reduced binding to c-di-AMP with a *K*_d_ of 7 *μ*mol/L. The N41A mutant had even weaker binding to c-di-AMP with a *K*_d_ of ∽140 *μ*mol/L, though saturation was not achieved in this binding titration, precluding an accurate measure of the affinity. These results confirm that the c-di-AMP binding site identified by structural analysis is the true binding site in solution.

### Conformations of the c-di-AMP molecules

**Figure 4:**
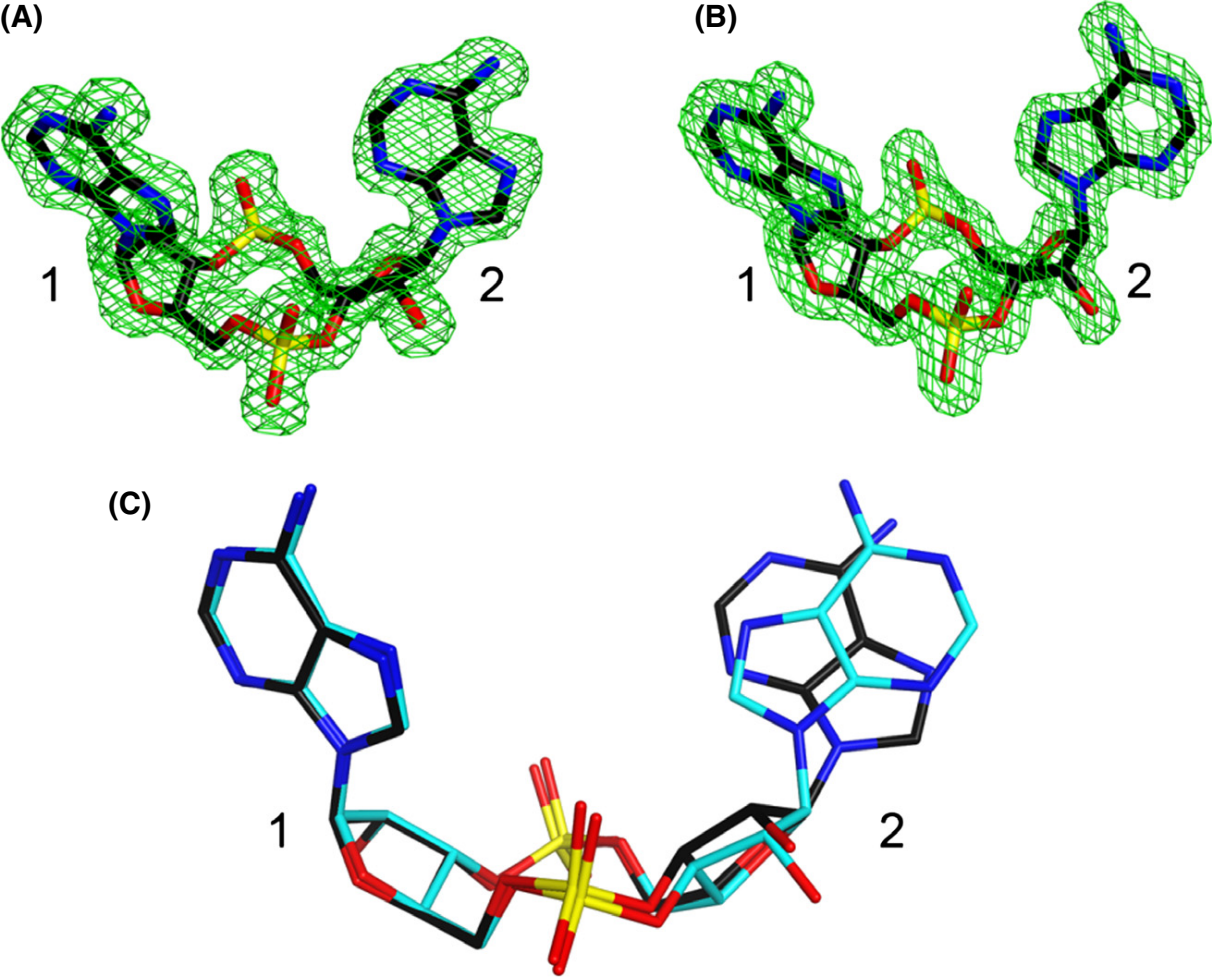
Conformations of the cyclic-di-AMP (c-di-AMP) molecules. (A) Omit F_o_–F_c_ electron density for c-di-AMP with the second nucleotide in the *syn* conformation at 1.6 Å resolution, contoured at 3*σ*. The first and second nucleotides are labeled. (B) Omit F_o_–F_c_ electron density for c-di-AMP with the second nucleotide in the *anti* conformation at 1.6 Å resolution, contoured at 3*σ*. (C) Overlay of two c-di-AMP molecules, with the second nucleotide in *syn* or *anti* conformation.

The adenine base of the first nucleotide in all three c-di-AMP molecules is in the *anti* conformation (Fig.3B), as is the case with ATP bound to P_II_. This is consistent with the several specific interactions the adenine has with PstA. In comparison, the adenine base for the second nucleotide in two of the c-di-AMPs assumes a *syn* conformation while the third one has an *anti* conformation (Fig.4A and B). In both conformations, the adenine base is *π*-stacked against the Phe36 side chain. The ability of this adenine to adopt *syn* and *anti* conformations is consistent with the relative weak interactions it has with PstA. It is unclear if this conformational flexibility has any functional relevance.

In the structure of the *ydaO* riboswitch in complex with c-di-AMP, the c-di-AMP molecules also have one adenine in *syn* and one in *anti* (Gao and Serganov 2014; Jones and Ferre-D'Amare 2014; Ren and Patel 2014). All the adenines in the *anti* conformation have the C3′ endo pucker for its associated ribose, while those in the *syn* conformation have a C2′ endo ribose pucker (Fig.4C).

### Structural changes in PstA upon c-di-AMP binding

**Figure 5:**
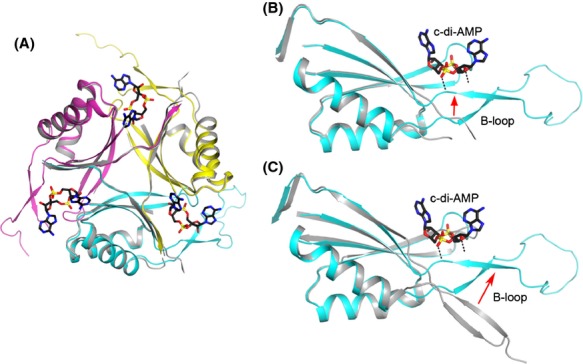
Comparisons between c-di-AMP bound and apo forms of LmPstA. (A) Overlay of cyclic-di-AMP (c-di-AMP) bound and apo forms of the LmPstA trimer. (B) Overlay of an LmPstA monomer with c-di-AMP bound (cyan) and in the apo (gray) form. The red arrow highlights the conformational changes of the B-loop upon c-di-AMP binding. (C) Overlay of an LmPstA monomer with c-di-AMP bound (cyan) and a monomer of the apo form of *Pediococcus pentosaceus* PstA (gray).

The overall structure of the c-di-AMP-bound form of the LmPstA trimer is almost identical to the apo structure with an r.m.s. distance of 0.6 Å for their C*α* atoms when they are overlaid (Fig.5A). However, there are significant differences in the conformations of the T-loop and B-loop between the apo and c-di-AMP-bound structures. In the apo LmPstA structure, the T-loop from residues 33–39 are largely disordered, indicating that this loop is flexible in the absence of c-di-AMP binding. In addition, the B-loop kinks at a conserved 94-GGA-96 motif upon c-di-AMP binding significantly change the location and orientation of the loop (Fig.5B, Video S1). Several interactions between LmPstA and c-di-AMP mediate the movement of the B-loop upon ligand binding. The main chain nitrogen of Gly94 interacts with the phosphate of the first nucleotide of c-di-AMP, and the main chain carbonyl of Gln92 interacts with the 2′ OH of the second nucleotide.

The C-terminal His-tag of the apo LmPstA structure forms *β*-sheet interactions with the B-loop, possibly affecting its conformation. Thus, we also compared the c-di-AMP-bound LmPstA with the apo form of a PstA homolog from *P. pentosaceus* which was crystallized with an N-terminal His-tag (Fig.5C). The same differences in the B-loop are also seen in this comparison, and because the B-loops are more ordered in the *P. pentosaceus* structure, the conformational changes that occur upon c-di-AMP binding are even more apparent.

While our article was under preparation and review, the structures of PstA from *S. aureus* (SaPstA) (Campeotto et al. 2015; Muller et al. 2015) and a PstA homolog DarA from *Bacillus subtilis* (Gundlach et al. 2015) bound to c-di-AMP were reported. The detailed interactions with c-di-AMP for SaPstA and DarA are largely identical to what we observed for LmPstA. The main difference in the ligand binding mode between these structures and LmPstA is that the second adenine of c-di-AMP bound to SaPstA and DarA is sandwiched between the side chains of Phe36 and Arg26, forming *π*-stacking and cation–*π* interactions. This Arg26 residue is present in most PstA proteins (Fig.1A), but in LmPstA this residue is instead a glycine and the cation–*π* interaction is absent. This indicates that the arginine side chain is dispensable for c-di-AMP binding. In addition, the c-di-AMP molecules in the SaPstA and DarA structures all adopt an *anti* conformation, in contrast to the LmPstA structure. The presence of the Arg26 residue may promote the *anti* conformation of the adenine base.

The conformational change of the B-loop upon c-di-AMP binding that was observed for LmPstA is also present in DarA, but is absent in the Muller et al. (2015) SaPstA structure. The Campeotto et al. (2015) SaPstA structure shows one B-loop with a conformational change, while this change is absent in the other two B-loops. These differences in the B-loop conformations may be due to crystal-packing influence in the SaPstA structures, in which the N-terminal His-tag of an adjacent SaPstA trimer makes several interactions with the c-di-AMP molecule in place of the B-loop.

### Functional studies of PstA

**Figure 6:**
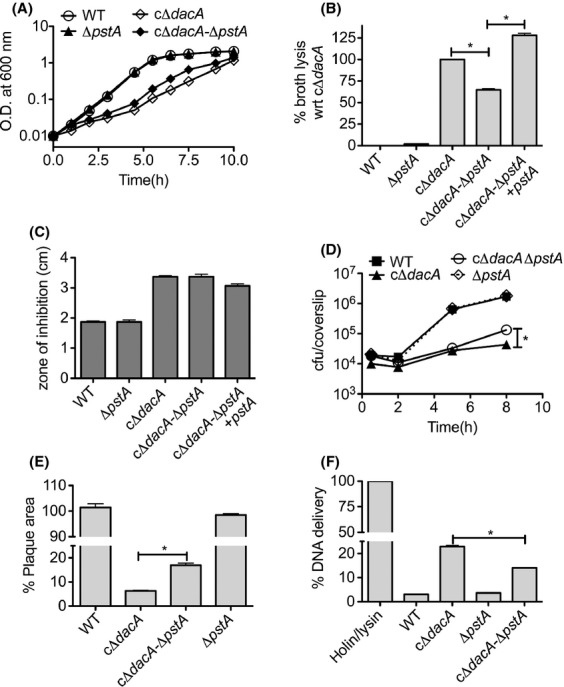
PstA functional studies in *Listeria* monocytogenes. (A) Growth of *L. monocytogenes* strains in Brain-heart infusion (BHI) media. (B) *β*-galactosidase release assay during mid-exponential growth of indicated *L. monocytogenes* strains. Percent bacteriolysis is normalized with respect to the cΔ*dacA* strain. (C) Kirby–Bauer disk diffusion assay measuring the zone of inhibition around a filter disk impregnated with 20 *μ*g of cefuroxime antibiotic. Values are reported as the diameter of the zones of inhibition. (D) Immortalized bone marrow-derived macrophages (iBMMs) were infected with indicated *L. monocytogenes* strains and CFU (colony forming units) were enumerated at various times post infection. (E) Plaque area from mouse fibroblasts (L2 cells) infected with indicated strains for three days and normalized to wild type (WT). (F) Intracellular lysis of bacterial strains in iBMMs as measured by reporter plasmid delivery. Percent lysis was determined by normalizing to Holin–Lysin and uninfected controls. For all panels, the data are the mean ± SEM of at least three measures and are representative of multiple independent experiments (*n *≥* *2). *P ≤ 0.01 by two-tailed *t*-test.

To interrogate the role of PstA-mediated c-di-AMP signaling in *L. monocytogenes*, we generated Δ*pstA* clean deletions in both WT and a conditional depletion strain of the sole diadenylate cyclase *dacA* (cΔ*dacA*) (Witte et al. 2013) (Tables3 and 4). Previous studies have shown that lowering the c-di-AMP level in *L. monocytogenes* causes a severe growth defect in nutritive media, defects in intracellular growth, and enhanced bacteriolysis in these environments. The absence of *pstA* in the cΔ*dacA* strain resulted in a slightly improved growth rate, with the cΔ*dacA* Δ*pstA* mutant exhibiting a 70-min doubling time, while cΔ*dacA* exhibited an 80-min doubling time. The Δ*pstA* mutant had no effect in the WT parental background (Fig.6A). Bacteriolysis measured as *β*-Gal release into the culture supernatant also revealed a partial rescue of the cell lysis defect in the cΔ*dacA* strain, with the double cΔ*dacA*-Δ*pstA* mutant exhibiting a twofold reduction relative to the parental strain alone (Fig.6B). Introduction of *pstA* on a plasmid with a constitutively active promoter (P_SPAC_) resulted in enhanced broth lysis beyond levels observed in the cΔ*dacA* strain, likely as a result of over complementation of PstA levels in this strain. In contrast to our expectation, there was no change in cefuroxime sensitivity for cΔ*dacA* strain after deletion of *pstA* (Fig.6C), suggesting that the stabilization of bacterial cell after *pstA* deletion in the cΔ*dacA* strain may not be directly linked to a weakened cell wall.

We next assessed the effect of PstA on intracellular growth in macrophages and fibroblasts. In accordance with the broth growth, deletion of *pstA* had no effect on the growth of the WT strain but resulted in a small but reproducible effect on the growth defect exerted by loss of c-di-AMP production. Specifically, the double cΔ*dacA*-Δ*pstA* mutant was observed to reach slightly higher levels of intracellular growth after 8 hours of BMM infection (Fig.6D). To examine the effect of PstA over an extended infection period, fibroblast plaque assays were performed for 3 days. In accordance with macrophage growth, the plaques formed by the double cΔ*dacA*-Δ*pstA* exhibited a twofold increase in area relative to the cΔ*dacA* strain (Fig.6E). We previously showed that the cΔ*dacA* mutant undergoes enhanced lysis in macrophages (Witte et al. 2013; Sureka et al. 2014). Therefore, we tested the intracellular lysis of *pstA* mutants by a DNA delivery assay (Sauer et al. 2010). The results showed a twofold reduction in cell lysis in the cΔ*dacA-*Δ*pstA* double mutant compared to cΔ*dacA*, whereas the Δ*pstA* remained unaffected (Fig.6F). Together these studies suggest that in the absence of c-di-AMP production, PstA has a negative impact on bacterial growth and survival both in and out of the host and that these are likely due to effects on bacterial cell stability.

**Table 3:** Strains used in this study

Strain	Strain number	Description	Reference
WT Lm	JW6	*Listeria monocytogenes* 10403s	
cΔ*dacA*	JW102	Conditional depletion strain of *dacA*	Witte et al. (2013)
*ΔpstA*	JW264	*pstA* deletion mutant	This study
cΔ*dacA*-ΔpstA	JW282	Strain 102 with deletion in *pstA*	This study
cΔ*dacA*-ΔpstA pBAV1K-*Pspac*-hy-*pstA*	JW457	Complementation of *pstA* disruption in strain 282	This study
Holin–Lysin	JW128	Strain expressing phage holin and lysin driven by the cytosol-specific *actA* promoter	Sauer et al. (2010)

**Table 4:** Plasmids used in this study

Strain number	Description	Reference
JW219	*pstA* in pET20b	This study
JW458	*pstA* F36A in pET20b	This study
JW459	*pstA* N41A in pET20b	This study
JW460	*pstA* under control of hyper-*Pspac* promoter in pBAV1K plasmid	This study

## Discussion

### Sequence conservation

**Figure 7:**
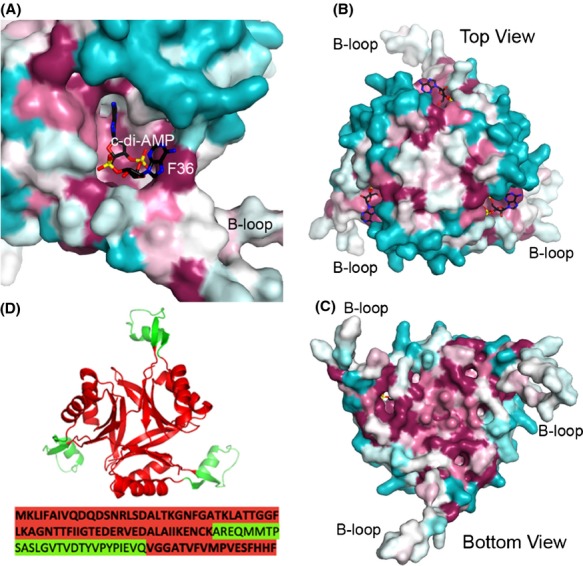
Sequence conservation of PstA. (A) Sequence conservation of residues in the cyclic-di-AMP (c-di-AMP) binding site, generated based on an alignment of 150 sequences by the program ConSurf (Armon et al. 2001). Purple indicates conserved residues, cyan indicates variable residues, and white indicates average conservation. The c-di-AMP molecule is labeled. (B) Top view of the LmPstA trimer showing sequence conservation. (C) Bottom view of the LmPstA trimer showing sequence conservation. (D) Mapping of LmPstA peptides identified by Sureka et al. (2014) to the structure. Peptides identified by quantitative shotgun sequencing are highlighted in red and those absent are in green.

Sequence conservation analysis of the PstA protein family shows that the residues identified in the structure as being important for c-di-AMP recognition are highly conserved (Fig.7A). These include the 34-GGFL-37 residues on the T-loop which interact with the second nucleotide of c-di-AMP. In addition, the 94-GGA-96 sequence at the B-loop kink which allows for the loop to move toward c-di-AMP is also universally conserved. The surface of the rest of the PstA trimer is less conserved with the exception of several residues on the bottom of the trimer which are likely important for the structural integrity of the trimer (Fig.7B and C). Unlike P_II_ proteins where the extended T-loop is highly conserved (Huergo et al. 2013), the extended B-loop in PstA has only weak sequence conservation (Fig.7B and C). Notably, Tyr51 on the T-loop of P_II_ proteins is strictly conserved and can be modified by reversible uridylylation or adenylation in some bacterial species (Huergo et al. 2013). P_II_ proteins interact with their protein targets primarily through T-loop-mediated interactions, and the uridyl-modification of Tyr51 has been shown to disrupt the interaction of the P_II_ protein GlnK with the ammonium channel AmtB (Conroy et al. 2007). The two tyrosine residues on the B-loop of PstA have only ∽70% sequence conservation (Fig.1A). Furthermore, *L. monocytogenes* does not contain a homolog of the *E. coli* uridylyl-transferase GlnD. However, we did note that within the shotgun sequencing that previously identified LmPstA as a c-di-AMP binding protein (Sureka et al. 2014), the peptides mapping to the B-loop of LmPstA were notably absent (Fig.7D), consistent with a modification to this region of the protein that precluded its identification. Together these observations provide intriguing albeit speculative evidence of a modification to this site. However, given the absence of GlnD homologs in the Firmicutes (Huergo et al. 2013), it is unlikely that if present this modification is uridylylation or adenylation of the B-loop tyrosine residues common to GlnD-mediated P_II_ modification and may be due to phosphorylation, as has been described in cyanobacteria (Forchhammer and Tandeau de Marsac 1995).

The core structure of P_II_ and PstA are remarkably similar (r.m.s. distance 0.9 Å for the C*α* atoms) despite only ∽15–20% sequence identity. In addition, the recognition of the equivalent adenine of c-di-AMP and ATP are almost identical in PstA and P_II_ proteins. The structures differ in the loop regions which are less important for the overall structural integrity of the trimer but are critical for defining the ligand binding site. Thus, it is likely that PstA and P_II_ arose from a common protein ancestor, and diverged through evolutionary changes in these loop regions. On the basis of these observations, we conclude that PstA is related to the larger P_II_ protein superfamily, but has diverged through evolution to specifically recognize the bacterial second messenger c-di-AMP and thus is structurally and functionally distinct from the canonical P_II_ proteins.

### Possible functions of PstA

PstA currently has no known function. Based on its structural similarity to P_II_ proteins, it is likely that PstA functions through a P_II_-like mechanism of binding and modifying the activities of its protein targets. There have been several reports of P_II_ proteins bound to various macromolecules. The stoichiometry of these complexes are either one P_II_ trimer bound to a trimeric protein in the case of AmtB and *N*-acetylglutamate kinase (Conroy et al. 2007; Gruswitz et al. 2007; Llacer et al. 2007; Mizuno et al. 2007), or to three separate monomers in the case of PipX and DraG (Llacer et al. 2010; Rajendran et al. 2011). In almost all of these complexes, the T-loop plays a critical role in complex formation, and is found in a different conformation in each complex. A notable exception is the P_II_–DraG complex where the T-loop is not involved in binding DraG, which instead binds to the lateral face of the P_II_ trimer close to the ADP binding site. In the case of AmtB and *N*-acetylglutamate kinase, ATP binding to P_II_ promotes complex formation through a mechanism involving changes in the T-loop conformation (Durand and Merrick 2006; Llacer et al. 2007). In Mtb P_II_, the T-loop makes several contacts with ATP, supporting such a mechanism (Shetty et al. 2010). Thus, ligand binding induces a particular shape and orientation of the T-loop in P_II_ which plays a key role in complex formation with its protein target.

Although we provide indications that PstA contributes to bacterial cell stability in the absence of c-di-AMP production, the identity of protein regulatory targets, which parts of the PstA trimer are involved in target recognition, and the cellular consequences of target recognition remain to be discovered. The genetic context of c-di-AMP riboswitches and the posttranslational effects on protein targets support a general role for c-di-AMP in osmotic stress responses in Firmicutes, within which PstA homologs are conserved (Corrigan et al. 2013; Nelson et al. 2013; Sureka et al. 2014). Consistent with these observations, our findings link PstA-mediated c-di-AMP signaling to bacteriolysis. If canonical P_II_ protein regulatory mechanisms provide any insight into the biological role of PstA, it is feasible that PstA may regulate membrane transporter function analogous to GlnK regulation of the ammonium transporter. Such transporters may be linked to ion or osmolyte levels that help to stabilize the cell. Conversely, metabolic effects reminiscent of P_II_ regulation of glutamine synthetase may impose changes in cell wall synthesizing capacity or osmolyte synthesis to affect cell stability.

Based on the large movement of the B-loop upon c-di-AMP binding, which dramatically changes the overall shape of the PstA trimer, it is likely that c-di-AMP binding can modulate the binding of PstA to its target proteins. However, the B-loop is significantly longer and less conserved than the P_II_ T-loop, so any protein–protein interactions mediated by the B-loop are likely to be different than what has been observed for P_II_ proteins. Clearly, identification and characterization of the binding partners of PstA will reveal significant insight into the mechanism by which PstA-mediated c-di-AMP signaling affects the physiology and cellular stability within the Firmicutes.

## Supporting Information

Video S1 Conformational changes of PstA upon c-di-AMP binding
